# Factors for the development of anemia in patients with newly introduced olaparib: A retrospective case-control study

**DOI:** 10.1097/MD.0000000000034123

**Published:** 2023-07-28

**Authors:** Chihiro Shiraishi, Toshinori Hirai, Michiko Kaneda, Akiharu Okamoto, Hideo Kato, Kayo Tanaka, Eiji Kondo, Tomoaki Ikeda, Takuya Iwamoto

**Affiliations:** a Department of Pharmacy, Mie University Hospital, Mie, Japan; b Department of Obstetrics and Gynecology, Mie University School of Medicine, Mie, Japan.

**Keywords:** anemia, folate, mean corpuscular volume, nutrition, olaparib

## Abstract

Anemia is the most common dose-limiting toxicity of olaparib. However, few studies have analyzed the clinical features of olaparib-induced anemia. This study investigated the clinical features of olaparib-induced anemia. Additionally, the role of folate or vitamin B_12_ in olaparib-induced anemia was examined. This retrospective case-control study included patients who received olaparib at Mie University Hospital between January 2018 and December 2020. Data were collected between initiation of olaparib and discontinuation of olaparib or till December 2021. We investigated the development of grade ≥ 3 anemia during olaparib administration for at least 1 year. We examined patients with grade ≥ 3 anemia considering the mean corpuscular volume (MCV), its association with gastrointestinal events and cumulative dose of carboplatin. For the sub-study analysis, data on patients treated with olaparib for ovarian or endometrial cancer were collected to evaluate the Common Terminology Criteria for Adverse Events (CTCAE) or monthly changes in folate or vitamin B_12_ levels from baseline to 3 months after olaparib initiation. These data were collected between initiation of olaparib and discontinuation of olaparib or till November 2022. Patients with no data on folic acid or vitamin B_12_ levels were excluded from the sub-study. In the main study, 40 patients were included. Eighteen patients (45%) developed grade ≥ 3 anemia, and all patients discontinued treatment (94%) or reduced olaparib dose (67%) after developing anemia. Among the patients with grade ≥ 3 anemia, 9 (50%) exhibited macrocytic anemia and 15 (83%) had previously received carboplatin. The incidence of grade ≥ 2 dysgeusia was significantly higher in patients with grade ≥ 3 anemia (*P* = .034). Moreover, the cumulative dose of previously administered carboplatin was higher in patients who had 3 episodes of anemia (*P* = .102). In sub-study, 12 had data on folic acid and vitamin B_12_ levels. Sub-study analysis showed that none fulfilled the criteria for deficiency of folate or vitamin B_12,_ while 3 developed grade 3 anemia. This study revealed that olaparib-induced anemia frequently occurs as macrocytic and normocytic erythroblastic anemia without folate or vitamin B_12_ deficiencies. A high cumulative dose of previously administered carboplatin and dysgeusia may be associated with olaparib-induced anemia.

## 1. Introduction

Olaparib is the first oral poly-ADP ribose polymerase (PARP) inhibitor used for the treatment of germline breast cancer susceptibility to gene (*BRCA*)-mutated cancers, such as ovarian cancer.^[1]^ Olaparib inhibits PARP and is involved in several processes including the repair of single-strand DNA breaks, genomic stability, and programmed cell death.^[2,3]^ Olaparib is a therapeutic option for maintenance therapy of ovarian cancer after platinum-based chemotherapy, regardless of the *BRCA* status.^[4]^

A phase III trial reported that olaparib-induced anemia was observed in 59.1% of patients (grade ≥ 3 anemia; 36.4%).^[5]^ Anemia is a dose-limiting toxicity.^[1]^ Grade ≥ 3 anemia potentially leads to treatment interruption until the recovery of hemoglobin (Hb) level ≥ 9.0 g/dL up to 4 weeks. Multiple and long-term withdrawals of olaparib often occur because of grade ≥ 3 anemia.^[1]^ Severe anemia requires transfusion of red blood cells (RBC) transfusion.^[6]^ In clinical practice, cancer-related anemia can be caused by several overlapping mechanisms (e.g., impaired erythropoiesis and chemotherapy-induced loss of appetite).^[7–9]^ For a more complete approach, careful evaluation of cancer-related anemia is important.

Several reports have demonstrated that folate deficiency is the main pathophysiological factor for olaparib-induced anemia irrespective of vitamin B_12_ deficiency.^[10,11]^ A case series study reported that severe folic acid deficiency occurred during olaparib therapy^[10]^; however, this study recruited patients who had developed severe folic deficiency at baseline. In addition, Yohannan reported only 1 case of severe folic acid deficiency during olaparib therapy.^[11]^ For these studies, variation factors of anemia in terms of changes in folate or vitamin B_12_ levels were not obtained.

In this study, we show the clinical features of olaparib-induced anemia in terms of changes in folate or vitamin B_12_ levels, co-administered chemotherapeutic agents, and gastrointestinal symptoms. Moreover, we present trends in monthly folic acid and vitamin B_12_ values, regardless of the onset of olaparib-related anemia.

### 1.1. Aim

In the present study, we retrospectively analyzed the subtypes of anemia associated with olaparib, the impact of chemotherapy history, and the details of olaparib treatment (treatment duration and adverse gastrointestinal events). Furthermore, we investigated the relationship between olaparib-induced anemia and changes in folate and vitamin B_12_ levels.

### 1.2. Ethics approval

This study was conducted in accordance with the Declaration of Helsinki and its amendments, after obtaining approval from the Clinical Research Ethics Review Committee of Mie University Hospital (main study: No. H2021-184 and sub-study: H2021-219). The requirement for patient consent was waived by the board because of the retrospective nature of the study.

## 2. Methods

### 2.1. Patients

This retrospective case-control study included data from ovarian, breast, endometrial, prostate, and pancreatic cancer patients aged ≥ 18 years who were administered olaparib at Mie University Hospital between January 2018 and December 2020. Data were collected between initiation of olaparib and discontinuation of olaparib or till December 2021. In the sub-study, data on folic acid and vitamin B_12_ levels of patients who received olaparib for the treatment of ovarian or endometrial cancer at Mie University Hospital between January 2018 and August 2022. Data were collected between initiation of olaparib and discontinuation of olaparib or till November 2022. In the main study, we did not set the exclusion criteria for the preliminary analysis. Patients without folic acid or vitamin B_12_ levels were excluded from the sub-study.

### 2.2. Data collection

Electronic medical charts of the patients were reviewed, and data were collected. We extracted demographic data (age, sex, height, body weight, body mass index, history of peptic ulcer, history of smoking, Brinkman index, and history of alcohol consumption), details of olaparib therapy (daily dose, and administration period), gene polymorphism and details of cancer (*BRCA* mutation status, and primary tumor location), previous treatment history (previous carboplatin treatment history, cumulative dose of carboplatin, radiation therapy, and RBC transfusion treatment), clinical laboratory data (serum albumin [Alb], C-reactive protein, blood urea nitrogen, serum creatinine [Scr], creatinine clearance [Ccr], estimated glomerular filtration rate [eGFR], lactate dehydrogenase, aspartate aminotransferase, alanine aminotransferase, total bilirubin, white blood cell, neutrophil, lymphocyte, platelet, RBC, Hb, hematocrit, mean corpuscular volume [MCV], mean corpuscular Hb, mean corpuscular Hb concentration, C-reactive protein/Alb ratio, red cell distribution width-standard deviation [RDW-SD], red cell distribution-coefficient of variation, folic acid, and vitamin B_12_ level). We also collected details on concomitant medications known to reduce folic acid levels (trimethoprim/sulfamethoxazole, methotrexate, antiepileptic drugs, and sulfasalazine)^[12–14]^ and vitamin B_12_ (proton pump inhibitor, histamine2 receptor antagonist, and metformin).^[12,15]^ Furthermore, we collected data on the concomitant use of medications, including strong cytochrome P450 3A4 (CYP3A4) inhibitors (clarithromycin, erythromycin, itraconazole, ketoconazole, and voriconazole), moderate CYP3A4 inhibitors (aprepitant, cimetidine, cyclosporin, fluconazole, fluvoxamine, imatinib, posaconazole, and verapamil), and CYP3A4 inducers (bosentan, carbamazepine, phenytoin, and rifampicin)^[16]^ because olaparib is primarily metabolized by CYP3A4/5 enzymes (84% of total clearance).^[17]^ The Brinkman index is a measure of cigarette smoke exposure and is calculated as the number of cigarettes smoked per day multiplied by the number of years of smoking.^[18]^ Renal function was evaluated using the Cockcroft-Gault equation: Ccr (mL/min) = (140–age) × body weight (× 0.85 if female)/(72 × Scr).^[19]^ eGFR was calculated using the Japanese GFR prediction equation: eGFR (mL/min/1.73 m^2^) = 194 × Scr^−1.094^ × age^−0.287^ (× 0.739 if female).^[20]^

### 2.3. Outcome

The primary outcome was grade ≥ 3 anemia (Hb < 8.0 g/dL) based on the Common Terminology Criteria for Adverse Events (CTCAE) of the National Cancer Institute (Supplemental Data 1, http://links.lww.com/MD/J187).^[21]^ Factors of anemia associated with olaparib, impact of chemotherapy history, treatment duration, and occurrence of adverse gastrointestinal events were evaluated between patient with and without onset of grade ≥ 3 anemia (Hb < 8.0 g/dL). Furthermore, type of amenia was categorized into 3 groups based on MCV (microcytic, MCV ≤ 80 fL; normocytic, 80 < MCV ≤ 100 fL; and macrocytic anemia, 100 < MCV fL)^[22]^ and investigated the subtypes of anemia associated with olaparib. For the sub-study analysis in the patients, for measuring folic acid or vitamin B_12_ values, the CTCAE grade or monthly changes in folate or vitamin B_12_ levels were collected from baseline to 3 months after olaparib initiation, regardless of the onset of anemia. Folate or vitamin B_12_ deficiency was defined as folate deficiency, folic acid < 4 ng/mL (normal range, ≥4 ng/mL), and vitamin B_12_ deficiency (<180 pg/mL; normal range, 180–914 pg/mL).

### 2.4. Statistical analysis

Statistical analyses were performed using JMP Pro 16 statistical package (SAS Institute, Cary, NC). Statistical significance was set at *P* < .05. Categorical data were summarized as numbers (%) and analyzed using the chi-square test. Continuous data were presented as median (interquartile range [IQR]) and analyzed using the Mann–Whitney *U* test. Missing values were handled without correction. In addition, the association between clinical factors and olaparib-related anemia was evaluated using the odds ratio (OR) with 95% confidence interval (CI) using a univariate logistic regression analysis. We further compared clinical characteristics according to significant variables. Since this was a pilot study, we collected as much patient data as possible.

Data, including anemia onset, lowest Hb value, ΔHb (baseline Hb level—nadir Hb level), RDW-SD, and red cell distribution width coefficient of variation, were compared. We calculated the incidence of anemia and compared the features of the initial onset of anemia stratified by the MCV. Next, we compared the MCV values between the baseline and the date of anemia onset (days 1–30, 31–60, 61–90, and >90) using Wilcoxon signed-rank test. We evaluated the association between anemia and adverse events related to dietary intake (dysgeusia and anorexia) using CTCAE ver. 5.0 (Supplemental Data 1, http://links.lww.com/MD/J187).^[21]^ In addition, we analyzed the relationship between the number of grade ≥ 3 anemia events and cumulative dose of carboplatin. The association of CTCAE-graded anemia events with folic acid or vitamin B_12_ levels and their baseline and monthly changes was also evaluated using the Kruskal−Wallis test.

## 3. Results

### 3.1. Baseline characteristics of patients and response to anemia

Supplemental Data 2, http://links.lww.com/MD/J188 shows a flowchart of the patient selection process. The baseline characteristics of the patients are summarized in Table 1. Thirty-one patients (78%) had ovarian cancer. Eighteen (45%) patients were *BRCA* mutation-positive, whereas the *BRCA* mutation status of 22 (55%) was unknown. One patient (3%) was administered 150 mg olaparib twice per day due to severe anemia (Hb 7.8 g/dL), and 2 patients (5%) were administered 200 mg olaparib twice per day due to renal impairment (Ccr: 29.0 mL/min and 45.0 mL/min). Among 40 patients, 2 (5%) received co-administered drugs suppressing folic acid, and 4 (10%) received co-administered drugs suppressing vitamin B_12_. None of the patients received potent CYP3A4 inhibitors or inducers.

**Table 1:** Patients baseline characteristics.

Demographics	Anemia (n = 18)	Non-anemia (n = 22)	*P* value
	n (%)	n (%)	
Age*, yr	62.0 [56.0–71.5]	60.0 [51.0–69.0]	.30
Female	16 (88.9)	21 (95.5)	.11
Body weight*, kg	53.9 [45.7–64.2]	53.6 [46.9–56.9]	.66
BMI*, kg/m^2^	22.8 [19.0–26.1]	22.1 [19.7–23.9]	.68
History of peptic ulcer	1 (5.6)	1 (4.5)	1.000
Smoking history	7 (38.9)	1 (4.5)	.01
Brinkman index*	170.0 [60.0–1125.0]	100.0 [100.0–100.0]	.66
History of alcohol consumption	3 (16.7)	2 (9.1)	.64
Previous carboplatin treatment history	15 (83.3)	19 (86.4)	.79
Cumulative dose of carboplatin*, mg/m^2^	3529.2 [2612.3–6718.0]	4132.0 [3334.2–4605.1]	.89
Radiation therapy	3 (16.7)	5 (22.7)	.64
RBC transfusion treatment	6 (33.3)	14 (63.6)	.11
Daily olaparib dose			
600 mg/d	16 (88.9)	21 (95.5)	.58
400 mg/d	1 (5.5)	1 (4.5)	1.000
300 mg/d	1 (5.5)	0 (0.0)	.45
*BRCA* mutation status			
Mutated germline *BRCA*	12 (66.7)	6 (27.3)	.02
Unknown	6 (33.3)	16 (72.7)	.02
Primary tumor location			
Ovaries	14 (77.8)	17 (77.3)	.71
Breast	2 (11.1)	2 (9.1)	1.000
Endometrial	1 (5.6)	2 (9.1)	1.000
Prostate	1 (5.6)	0 (0.0)	.45
Pancreas	0 (0.0)	1 (4.5)	1.000
Clinical laboratory data			
Alb*, g/dL	4.2 [4.0–4.4]	4.4 [4.1–4.5]	.28
CRP*, mg/dL	0.0 [0.0–0.2]	0.0 [0.0–0.2]	.74
BUN*, mg/dL	14.7 [11.5–17.7]	14.9 [10.7–20.2]	.95
Scr*, mg/dL	0.6 [0.6–0.7]	0.6 [0.6–0.7]	.27
Ccr [Cockcroft–Gault]*, mL/min	79.3 [63.6–93.9]	85.9 [67.7–98.5]	.51
eGFR*, mL/min/1.73 m^2^	76.3 [64.2–90.9]	82.2 [66.8–90.2]	.45
LDH*, IU/L	211.5 [171.0–257.8]	188.5 [176.8–212.8]	.28
AST*, U/L	22.0 [17.0–25.5]	22.0 [18.5–27.0]	.62
ALT*, U/L	12.5 [10.3–18.0]	16.0 [14.3–25.8]	.07
T–Bil*, mg/dL	0.5 [0.4–0.7]	0.5 [0.4–0.7]	.62
WBC*, ×10^3^/μL	4.8 [3.8–5.7]	4.0 [3.2–5.3]	.15
Net*,/μL	2715.0 [2010.0–3627.5]	2480.0 [2065.0–3105.0]	.75
Lym*,/μL	1315.0 [1045.0–1550.0]	1170.0 [790.0–1700.0]	.69
Plt*, ×10^3^/μL	182.0 [159.5–236.5]	223.0 [175.5–239.5]	.39
RBC*, ×10^6^/μL	3.6 [2.9–3.9]	3.3 [2.8–3.8]	.55
Hb*, g/dL	11.1 [10.1–12.4]	11.3 [10.1–12.4]	.93
Ht*, %	34.2 [30.4–36.6]	33.8 [30.5–37.6]	.71
MCV*, fL	99.5 [93.2–105.2]	99.7 [96.7–105.0]	.31
MCH*, pg	32.1 [31.6–33.5]	33.3 [31.9–35.8]	.14
MCHC*, g/dL	32.8 [32.3–33.5]	33.2 [32.5–33.5]	.52
CRP/Alb ratio*, 10^–3^	0.0 [0.0–0.0]	0.0 [0.0–0.1]	.33
RDW-SD*, fL	50.2 [45.4–58.5]	52.6 [42.9–55.4]	.60
RDW-CV*, %	15.1 [13.9–17.6]	14.2 [12.7–15.2]	.17
Co-administered drugs			
Suppressing folic acid	0 (0.0)	2 (9.1)	.20
Suppressing vitamin B_12_	2 (11.1)	2 (9.1)	.83

Anemia was defined as CTCAE grade ≥ 3 anemia (Hb < 8.0 g/dL).^[21]^

The median cumulative dose of carboplatin was calculated for patients with a history of carboplatin treatment.

* Data are presented as the median [interquartile range].

Ccr (mL/min) = (140–age) × body weight (× 0.85 if female)/(72 × Scr).^[19]^

eGFR (mL/min/1.73 m^2^) = 194 × Scr^−1.094^ × age^−0.287^ (× 0.739 if female).^[20]^

Alb = serum albumin, ALT = alanine aminotransferase, AST = aspartate aminotransferase, BMI = body mass index, BRCA = tumor breast cancer susceptibility gene, BUN = blood urea nitrogen, CCr = creatinine clearance, CRP = C-reactive protein, CTCAE = Common Terminology Criteria for Adverse Events, eGFR = estimated glomerular filtration rate, Hb = hemoglobin, Ht = hematocrit, LDH = lactate dehydrogenase, Lym = lymphocyte, MCH = mean corpuscular hemoglobin, MCHC = mean corpuscular hemoglobin concentration, MCV = mean corpuscular volume, Net = neutrophil, Plt = platelet, RBC = red blood cell, RDW-CV = red cell distribution width coefficient of variation, RDW-SD = red cell distribution width standard deviation, Scr = serum creatinine, T-Bil = total bilirubin, WBC = white blood cell.

Fifteen (38%) and 3 (8%) patients developed grade 3 and 4 anemia, respectively. The incidence of anemia was significantly higher among patients with *BRCA* mutations (anemia, n = 12 [67%] vs non-anemia, n = 6 [27%]; *P = *.024) (OR = 5.3, 95% CI = 1.4–20.7, *P = *.01). Among the 8 patients with a history of smoking, 7 developed anemia, which was significantly higher than that in patients without a smoking history (*P = *.014) (OR = 13.4, 95% CI = 1.5–122.9, *P = *.02). The primary physician decided to discontinue the treatment (94%) or reduce the olaparib dose (67%) after the onset of olaparib-induced anemia. In addition, 6 patients (33%) received 2 to 4 IU RBC (RBC Leukocytes Reduced, NISSEKI), while 5 (28%) received oral iron preparations, as shown in Supplemental Data 3, http://links.lww.com/MD/J189.

### 3.2. Anemia-related laboratory data of patients during initial olaparib-induced anemia

The patient characteristics during the first onset of olaparib-induced anemia are shown in Supplemental Data 4, http://links.lww.com/MD/J190. Of the 18 patients who developed anemia, 9 (50%) developed macrocytic anemia with high RDW-SD values (MCV ≤ 100 fL, 50.7 [44.4–60.0] fL vs MCV > 100 fL, 65.3 [55.2–72.0] fL, *P* = .01) (OR = 1.2, 95% CI = 1.0–1.4, *P < *.001). None of the patients had microcytic anemia.

Supplemental Data 5, http://links.lww.com/MD/J191 shows the characteristics of the patients in the 2 groups divided by MCV at the onset of anemia. Approximately half of the patients who developed normocytic anemia had hypoalbuminemia (Alb < 4.0 g/dL) (80 < MCV ≤ 100 fL, 4 [44%] vs 100 < MCV fL, 0 [0%], *P* = .04).

### 3.3. Comparison of MCV between baseline and onset of anemia

**Figure 1.**
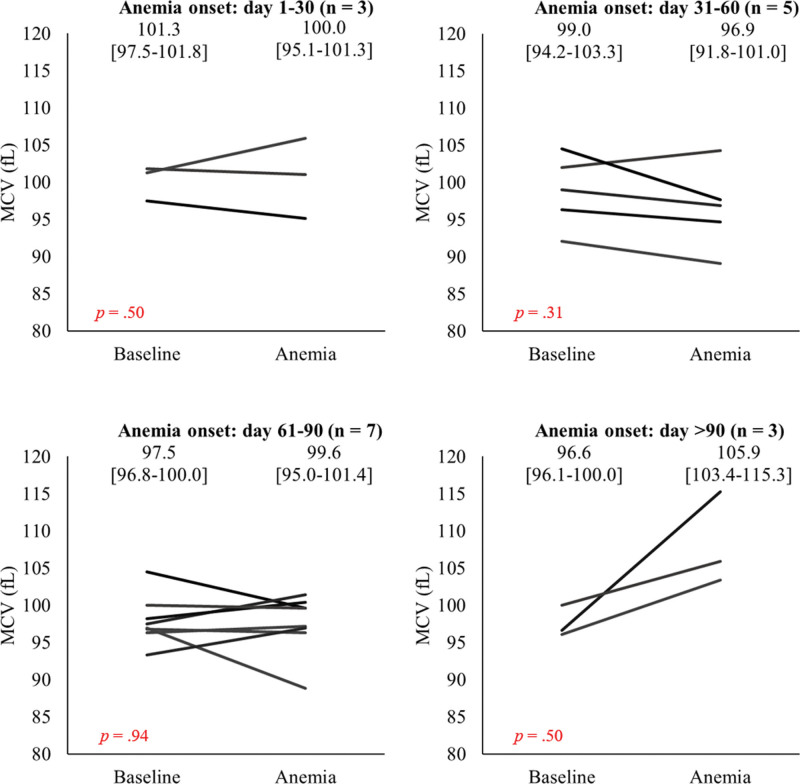
Comparison of MCV values between baseline and date of anemia onset (d 1–30, 31–60, 61–90, and >90). The X-axis represents the measurement time of MCV. The “baseline” indicates the day before initiating olaparib therapy and “anemia” indicates the onset of grade ≥3 anemia (Hb < 8.0 g/dL). The Y-axis represents MCV values. The boxplot indicates minimum, 25th percentile, median, 75th percentile, and maximum. The median (IQR) value is indicated in the upper side of the graph. If patients developed anemia several times, each nadir MCV values were counted as 1 case. Hb = hemoglobin, IQR = interquartile range, MCV = mean corpuscular volume.

Although no significant difference was observed between baseline MCV and MCV at the onset of anemia during the study period (Fig. 1), MCV values tended to be higher at the onset of anemia after 90 days than at baseline (baseline, 96.6 [96.1–100.0] fL vs > 90 days, 105.9 [103.4–115.3] fL, *P* = .05).

### 3.4. Occurrence of anemia and adverse events in relation to dietary intake

**Figure 2.**
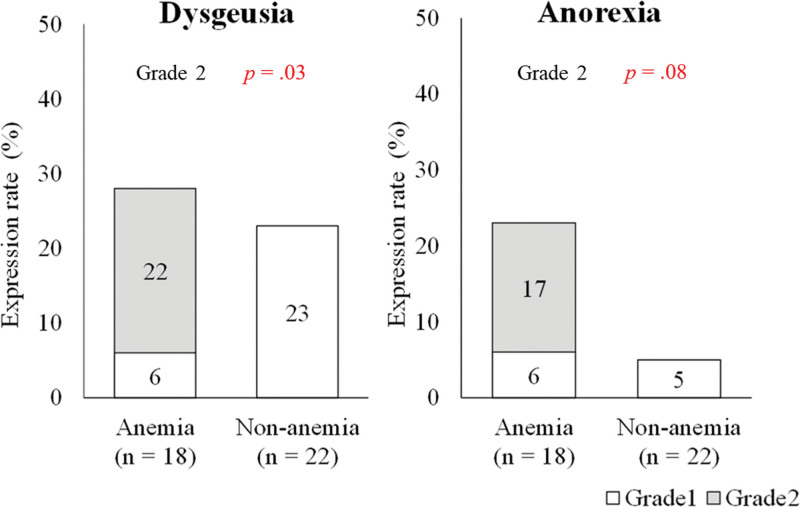
Association between anemia and adverse events related to dietary intake (dysgeusia and anorexia) based on CTCAE ver. 5.0. The X-axis represents the group classified by anemia onset, and the Y-axis represents the expression rate (%) of each grade (grades 1 and 2). CTCAE = Common Terminology Criteria for Adverse Events.

Patients who developed grade ≥ 3 anemia were more likely to experience grade ≥ 2 dysgeusia (anemia, 4/18 [22%] vs non-anemia, 0/22 [0%]; *P* = .03), as shown in Figure 2. Similarly, the rate of anorexia in patients with anemia was significantly higher than that in patients without anemia (anemia, 3/18 [17%] vs non-anemia, 0/22 [0%]; *P* = .08).

### 3.5. Relationship between cumulative dose of previous carboplatin and number of anemia events

**Figure 3.**
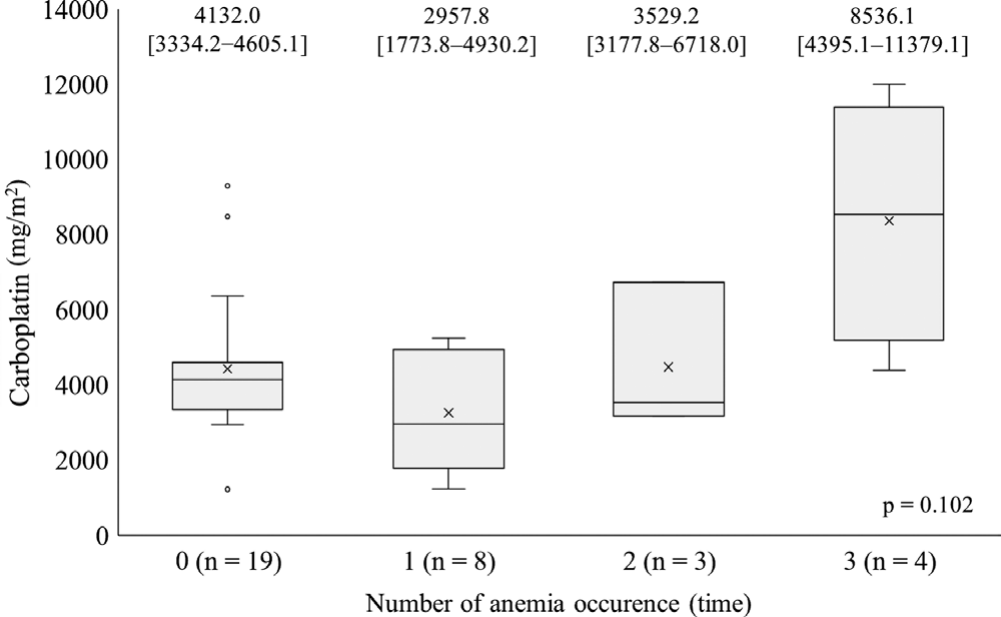
Relationship between cumulative dose of previous carboplatin and episode of anemia events. The X-axis represents episode of grade ≥3 anemia (Hb < 8.0 g/dL) events. The Y-axis represents cumulative dose of previous carboplatin (mg/m^2^). The boxplot indicates minimum, 25th percentile, median, 75th percentile, and maximum. The median (IQR) value is indicated in the upper side of the graph. Four patients who developed anemia 3 times had a relatively high cumulative dose of carboplatin (8536.1 [4395.1–11379.1] mg/m^2^). CTCAE = Common Terminology Criteria for Adverse Events, Hb = hemoglobin, IQR = interquartile range.

Of the 18 patients who developed anemia, 15 (83%) had a history of carboplatin administration. Although no significant difference was observed, the median cumulative dose of carboplatin in 4 patients with 3 episodes of anemia was relatively higher than that in patients with 2 or fewer episodes of anemia (8536.1 [4395.1–11379.1] mg/m^2^ *P* = .10) (Fig. 3).

### 3.6. Analysis of folic acid and vitamin B_12_ levels (Sub-study)

**Figure 4.**
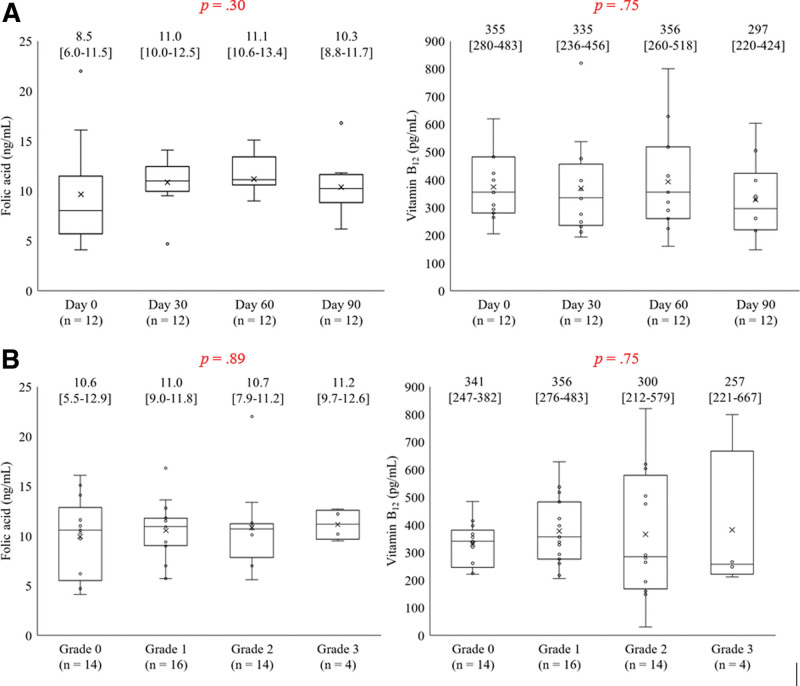
(A) Monthly changes in folic acid or vitamin B_12_ levels. Time-dependent folic acid or vitamin B_12_ level at baseline and every month for 3 mo were investigated. The X-axis represents administration d (d 0, 30, 60, and 90). The Y-axis represents folic acid or vitamin B_12_ value. The boxplot indicates minimum, 25th percentile, median, 75th percentile, and maximum. The median (IQR) value is indicated in the upper side of the graph. (B) CTCAE grading of anemia and folic acid or vitamin B_12_ levels. The relationship between CTCAE grading anemia onset and folic acid or vitamin B_12_ value at baseline and every month for 3 mo were investigated. The X-axis represents CTCAE grade of anemia during olaparib therapy. The Y-axis represents folic acid or vitamin B_12_ level. The boxplot indicates minimum, 25th percentile, median, 75th percentile, and maximum. The median (IQR) value is indicated in the upper side of the graph. CTCAE = Common Terminology Criteria for Adverse Events, IQR = interquartile range.

Among the 41 patients with ovarian or endometrial cancer who received olaparib, 12 had elevated folic acid and vitamin B_12_ levels. Supplemental Data 2, http://links.lww.com/MD/J188 shows a flowchart of the patient selection process. In the sub-study, the exclusion criteria were as follows: no measurement of folic acid or vitamin B_12_ levels (n = 29). The baseline characteristics of the patients are summarized in Table 2. None of the patients had folate or vitamin B12 deficiency with/without anemia onset. In addition, none of the patients had folate or vitamin B_12_ deficiency, regardless of the olaparib treatment duration or severity of anemia (Fig. 4A and B). Three patients (25%) had grade 3 anemia. It was noted that baseline low Hb cases developed grade 3 anemia following olaparib administration, regardless of the folic acid or vitamin B_12_ levels.

**Table 2:** Baseline characteristics of the patients whose folic acid and vitamin B_12_ levels were measured.

Demographics	n = 12
	n (%)
Age*, yr	65.5 [54.8–72.0]
Female	12 (100.0)
Body weight*, kg	54.0 [46.7–59.3]
BMI*, kg/m^2^	22.4 [18.7–23.2]
History of peptic ulcer	0 (0.0)
Smoking history	1 (8.3)
History of alcohol consumption	1 (8.3)
Previous carboplatin treatment history	12 (100.0)
Cumulative dose of carboplatin*, mg/m^2^	2805.4 [2484.8–3204.9]
Radiation therapy	0 (0.0)
RBC transfusion treatment	1 (8.3)
*BRCA* mutation status	
Mutated germline *BRCA*	3 (25.0)
Unknown	1 (8.3)
Primary tumor location	
Ovarie	9 (75.0)
Breast	0 (0.0)
Endometrial	3 (25.0)
Prostate	0 (0.0)
Pancreas	0 (0.0)
Clinical laboratory data	
Alb*, g/dL	4.2 [4.0–4.4]
CRP*, mg/dL	0.1 [0.0–0.1]
BUN*, mg/dL	14.3 [12.6–18.5]
Scr*, mg/dL	0.6 [0.5–0.7]
Ccr [Cockcroft–Gault]*, mL/min	79.5 [61.2–94.2]
eGFR*, mL/min/1.73 m^2^	77.1 [59.9–89.3]
LDH*, IU/L	197.0 [175.8–219.3]
AST*, U/L	21.0 [18.5–28.8]
ALT*, U/L	18.0 [15.0–22.3]
T–Bil*, mg/dL	0.7 [0.5–0.7]
WBC*, ×10^3^/μL	4.4 [3.6–5.3]
Net*,/μL	2165.0 [1915.0–2675.0]
Lym*,/μL	1386.0 [1152.5–1670.0]
Plt*, ×10^3^/μL	199.5 [138.8–233.5]
RBC*, ×10^6^/μL	3.1 [2.9–3.6]
Hb*, g/dL	10.4 [9.9–11.8]
Ht*, %	32.0 [30.1–36.2]
MCV*, fL	105.1 [100.1–107.2]
MCH*, pg	33.8 [33.1–34.4]
MCHC*, g/dL	32.4 [31.9–32.8]
CRP/Alb ratio*, ×10^–3^	0.0 [0.0–0.0]
RDW-SD*, fL	58.2 [52.7–65.4]
RDW-CV*, %	15.1 [14.1–16.5]
Co-administered drugs	
Suppressing folic acid	1 (8.3)
Suppressing vitamin B_12_	1 (8.3)

The median cumulative dose of carboplatin was calculated for patients with a history of carboplatin treatment.

* Data are presented as the median [interquartile range].

Ccr (mL/min) = (140–age) × body weight (× 0.85 if female)/(72 × Scr).^[19]^

eGFR (mL/min/1.73 m^2^) = 194 × Scr^−1.094^ × age^−0.287^ (× 0.739 if female).^[20]^

Alb = serum albumin, ALT = alanine aminotransferase, AST = aspartate aminotransferase, BMI = body mass index, BRCA = tumor breast cancer susceptibility gene, BUN = blood urea nitrogen, CCr = creatinine clearance, CRP = C-reactive protein, eGFR = estimated glomerular filtration rate, Hb = hemoglobin, Ht = hematocrit, LDH = lactate dehydrogenase, Lym = lymphocyte, MCH = mean corpuscular hemoglobin, MCHC = mean corpuscular hemoglobin concentration, MCV = = mean corpuscular volume, Net = neutrophil, Plt = platelet, RBC = red blood cell, RDW-CV = red cell distribution width coefficient of variation, RDW-SD = red cell distribution width standard deviation, Scr = serum creatinine, T-Bil = total bilirubin, WBC = white blood cell.

## 4. Discussion

Approximately half of the study population developed grade ≥ 3 anemia. The main types of anemia associated with olaparib were macrocytic and normocytic. The present study identified that a high cumulative dose of carboplatin and adverse events related to dietary intake, such as dysgeusia, may be associated with or accompanied by olaparib-induced anemia. However, none of the patients in the sub-study, even those with grade 3 anemia, developed severe folate or vitamin B_12_ deficiencies during olaparib therapy.

Notably, macrocytic anemia appeared to be a late-stage adverse effect of the olaparib treatment. Olaparib inhibits PARP-2 (50% inhibitory concentration of PARP-2:1–251 nM).^[23]^ In vivo studies have revealed that PARP-2 plays an essential role in the survival of hematopoietic stem/progenitor cells,^[24,25]^ indicating that loss of PARP-2 function leads to macrocytic anemia, impairing DNA metabolism, even with increased erythropoietin levels.^[25]^ Therefore, PARP-2 may be involved in the main mechanism of olaparib-induced anemia. The occurrence of olaparib-induced anemia reduces the clinical benefits and quality of life of the patients.^[26]^ Thus, clinicians must assess the characteristics and risk factors of olaparib-induced anemia.

Folate deficiency is a physiological condition that elevates MCV and red cell distribution values.^[27]^ Proton-coupled folate transporter, which is mainly located in intestinal epithelial cells, promotes the absorption of folic acid in conjunction with nuclear respiratory factor 1.^[28]^ For instance, proton-pump inhibitors have been shown to block the absorption of folic acid via the inhibition of proton-coupled folate transporter, thereby leading to macrocytic anemia.^[29]^ As PARP-1 plays an important role in transcriptional activation by nuclear respiratory factor-1,^[30]^ olaparib lowers intestinal folate absorption. However, none of the patients had folate or vitamin B_12_ deficiency during olaparib therapy, regardless of anemia grade according to the CTCAE (Fig. 4B). Folate deficiency is common in developing and Western countries.^[31]^ Further large-scale studies are necessary to obtain more robust evidence regarding the occurrence of olaparib-induced anemia.

Dysgeusia has been reported in approximately 20% of olaparib-treated patients with recurrent ovarian cancer.^[32]^ Regardless of the grade, dysgeusia has a significant effect on patients’ quality of life, which leads to loss of appetite.^[33]^ In our sub-study, none of the patients had folate or vitamin B_12_ deficiencies, even those with grade 3 anemia. However, hypoalbuminemia was often observed in patients with grade ≥ 3 anemia (*P* < .001) (data not shown), suggesting that severe anemia co-existed with malnutrition in these patients. Thus, clinicians must assess adverse events related to dietary intake to manage olaparib-induced anemia. Because there are no known standard pharmacological interventions for dysgeusia, its management should be tailored to an individual perceived taste changes.

Smoking causes inflammation, bone marrow suppression, and gastritis, resulting in anemia.^[34]^ Carbon monoxide binds to Hb in the blood to produce carboxyhemoglobin, which does not transport oxygen. Carboxyhemoglobin increases the production of erythrocytes, thereby upregulating apparent Hb.^[35]^ Although no differences were observed in the baseline Hb level regardless of smoking history, 7 (88%) of the 8 patients with a history of smoking developed anemia. Furthermore, smokers have also been reported to have a tendency to consume poorer diets than nonsmokers.^[36]^ In this study, the median Alb level was relatively low in the patients with a history of smoking. Based on these data, smoking history may be a possible risk factor for olaparib-induced anemia.

*BRCA* mutant cells have been reported to be more sensitive to olaparib treatment than other mutant cells.^[37]^ In addition, previous physiologically based pharmacokinetic modeling analysis has revealed that a 50% increase in exposure to olaparib would result in a ~17.5% decrease in Hb levels, indicating anemia, in *BRCA*-mutated ovarian cancer patients.^[17]^ Although 22 patients did not undergo *BRCA* mutation testing in our study, the incidence of anemia was significantly higher in patients with *BRCA* mutations, consistent with another report.^[38]^ Patients with *BRCA1/2* mutations are at risk of Fanconi anemia, which may affect the frequency and severity of olaparib-induced anemia.^[39,40]^ Chemotherapeutic agents can cause DNA damage and olaparib impedes repair.^[5,41]^ Carboplatin has been reported to exert antitumor effects through the formation of DNA adducts^[42]^ and may be associated with an increased risk of anemia.^[43]^ Additionally, bone marrow function deteriorates due to repeated chemotherapy cycles.^[44,45]^ Cancers arising from *BRCA* mutations are relatively hypersensitive to platinum-based therapies.^[46,47]^ Therefore, olaparib potentially contributes to the delayed recovery of blood cell counts in patients with a history of platinum treatment by inhibiting PARP-mediated DNA damage repair.

This study had several limitations. First, it was a retrospective study with a limited sample size; therefore, it was difficult to evaluate causality and avoid the impact of unknown confounding factors. Second, we could not obtain all the clinical data, such as *BRCA* mutation, or concomitant medication use. Finally, we could not measure diet, iron, and erythropoietin levels. Previous reports have revealed severe folic acid deficiency in patients with olaparib-induced anemia^[10,11]^; however, they did not analyze variation factors of anemia in terms of changes in folate or vitamin B_12_ levels. Our data failed to show the correlation between olaparib-induced anemia and folate or vitamin B_12_ deficiency, inconsistent with the previous studies. A multi-center prospective study should be conducted to validate the findings of olaparib-induced anemia, including genetic polymorphisms and concomitant medication use. A more detailed mechanism of olaparib-induced anemia will be determined through future animal experiments.

## 5. Conclusion

Our analysis showed that olaparib-induced anemia frequently occurs as macrocytic and normocytic erythroblastic anemia without folate or vitamin B_12_ deficiencies. In addition, a high cumulative dose of previously administered carboplatin and dysgeusia contributed to olaparib-induced anemia. Our findings are valuable for detecting olaparib-related anemia in clinical practice.

## Acknowledgments

We would like to thank Editage (www.editage.jp) for the English language editing.

## Author contributions

**Conceptualization:** Toshinori Hirai, Takuya Iwamoto.

**Formal analysis:** Chihiro Shiraishi, Toshinori Hirai.

**Investigation:** Chihiro Shiraishi.

**Methodology:** Hideo Kato, Toshinori Hirai.

**Resources:** Chihiro Shiraishi.

**Software:** Hideo Kato.

**Supervision:** Takuya Iwamoto.

**Writing – original draft:** Chihiro Shiraishi.

**Writing – review & editing:** Toshinori Hirai, Michiko Kaneda, Akiharu Okamoto, Hideo Kato, Kayo Tanaka, Eiji Kondo, Tomoaki Ikeda, Takuya Iwamoto.

## Correction

The first author’s name was originally listed incorrectly as Shiraishi Chihiro. It has been corrected to Chihiro Shiraishi throughout the article.

## Supplementary Material










