# Factors for the development of anemia in patients with newly introduced olaparib: A retrospective case-control study: Erratum

**DOI:** 10.1097/MD.0000000000039868

**Published:** 2024-11-08

In the article, “Factors for the development of anemia in patients with newly introduced olaparib: A retrospective case-control study,”^[1]^ which appears in Volume 102, Issue 30 of *Medicine*, the p-value appeared incorrectly for some rows in Table 1. The p-values originally appeared as <.001 and have been corrected to 1.000 in the following rows: History of peptic ulcer, Daily olaparid dose: 400 mg/d, and Breast, Endometrial, and Pancreas under Primary Tumor Location. Please see the correct Table 1 here:

**Table 1:** Patients baseline characteristics

Demographics	Anemia (n = 18)		Non-anemia (n = 22)	*p*–value
	n (%)		n (%)	
Age*, years	62.0 [56.0–71.5]		60.0 [51.0–69.0]	.30
Female	16 (88.9)		21 (95.5)	.11
Body weight*, kg	53.9 [45.7–64.2]		53.6 [46.9–56.9]	.66
BMI*, kg/m^2^	22.8 [19.0–26.1]		22.1 [19.7–23.9]	.68
History of peptic ulcer	1 (5.6)		1 (4.5)	1.00
Smoking history	7 (38.9)		1 (4.5)	.01
Brinkman index*	170.0 [60.0–1125.0]		100.0 [100.0–100.0]	.66
History of alcohol consumption	3 (16.7)		2 (9.1)	.64
Previous carboplatin treatment history	15 (83.3)		19 (86.4)	.79
Cumulative dose of carboplatin*, mg/m^2^	3529.2 [2612.3–6718.0]		4132.0 [3334.2–4605.1]	.89
Radiation therapy	3 (16.7)		5 (22.7)	.64
RBC transfusion treatment	6 (33.3)		14 (63.6)	.11
**Daily olaparib dose**				
600 mg/day	16 (88.9)		21 (95.5)	.58
400 mg/day	1 (5.5)		1 (4.5)	1.00
300 mg/day	1 (5.5)		0 (0.0)	.45
***BRCA* mutation status**				
Mutated germline *BRCA*	12 (66.7)		6 (27.3)	.02
Unknown	6 (33.3)		16 (72.7)	.02
**Primary tumor location**				
Ovaries	14 (77.8)		17 (77.3)	.71
Breast	2 (11.1)		2 (9.1)	1.00
Endometrial	1 (5.6)		2 (9.1)	1.00
Prostate	1 (5.6)		0 (0.0)	.45
Pancreas	0 (0.0)		1 (4.5)	1.00
**Clinical laboratory data**				
Alb*, g/dL	4.2 [4.0–4.4]	4.4 [4.1–4.5]		.28
CRP*, mg/dL	0.0 [0.0–0.2]	0.0 [0.0–0.2]		.74
BUN*, mg/dL	14.7 [11.5–17.7]	14.9 [10.7–20.2]		.95
Scr*, mg/dL	0.6 [0.6–0.7]	0.6 [0.6–0.7]		.27
Ccr [Cockcroft–Gault] *, mL/min	79.3 [63.6–93.9]	85.9 [67.7–98.5]		.51
eGFR*, mL/min/1.73m^2^	76.3 [64.2–90.9]	82.2 [66.8–90.2]		.45
LDH*, IU/L	211.5 [171.0–257.8]	188.5 [176.8–212.8]		.28
AST*, U/L	22.0 [17.0–25.5]	22.0 [18.5–27.0]		.62
ALT*, U/L	12.5 [10.3–18.0]	16.0 [14.3–25.8]		.07
T–Bil*, mg/dL	0.5 [0.4–0.7]	0.5 [0.4–0.7]		.62
WBC*, ×10^3^/μL	4.8 [3.8–5.7]	4.0 [3.2–5.3]		.15
Net*,/μL	2715.0 [2010.0–3627.5]	2480.0 [2065.0–3105.0]		.75
Lym*,/μL	1315.0 [1045.0–1550.0]	1170.0 [790.0–1700.0]		.69
Plt*, ×10^3^/μL	182.0 [159.5–236.5]	223.0 [175.5–239.5]		.39
RBC*, ×10^6^/μL	3.6 [2.9–3.9]	3.3 [2.8–3.8]		.55
Hb*, g/dL	11.1 [10.1–12.4]	11.3 [10.1–12.4]		.93
Ht*, %	34.2 [30.4–36.6]	33.8 [30.5–37.6]		.71
MCV*, fL	99.5[93.2–105.2]	99.7 [96.7–105.0]		.31
MCH*, pg	32.1 [31.6–33.5]	33.3 [31.9–35.8]		.14
MCHC*, g/dL	32.8 [32.3–33.5]	33.2 [32.5–33.5]		.52
CRP/Alb ratio*, 10^–3^	0.0 [0.0–0.0]	0.0 [0.0–0.1]		.33
RDW-SD*, fL	50.2 [45.4–58.5]	52.6 [42.9–55.4]		.60
RDW-CV*, %	15.1 [13.9–17.6]	14.2 [12.7–15.2]		.17
**Co-administered drugs**				
Suppressing folic acid	0 (0.0)	2 (9.1)		.20
Suppressing vitamin B_12_	2 (11.1)	2 (9.1)		.83

Anemia was defined as CTCAE grade ≥3 anemia (Hb <8.0 g/dL) ^21^

The median cumulative dose of carboplatin was calculated for patients with a history of carboplatin treatment.

* Data are presented as the median [interquartile range].

Ccr (mL/min) = (140–age) × body weight (× 0.85 if female)/(72 × Scr) ^19^

eGFR (mL/min/1.73 m^2^) = 194 × Scr^−1.094^ × age^−0.287^ (× 0.739 if female) ^20^

Alb = serum albumin, ALT = alanine aminotransferase, AST = aspartate aminotransferase, BMI = body mass index, *BRCA* = tumor breast cancer susceptibility gene, BUN = blood urea nitrogen, CCr = creatinine clearance, CRP = C-reactive protein, CTCAE = Common Terminology Criteria for Adverse Events, eGFR = estimated glomerular filtration rate, Hb = hemoglobin, Ht = hematocrit, LDH = lactate dehydrogenase, Lym = lymphocyte, MCH = mean corpuscular hemoglobin, MCHC = mean corpuscular hemoglobin concentration, MCV = mean corpuscular volume, Net = neutrophil, Plt = platelet, RBC = red blood cell, RDW-SD = red cell distribution width standard deviation, RDW-CV = red cell distribution width coefficient of variation, Scr = serum creatinine, T-Bil = total bilirubin, WBC = white blood cell
