# Supplementary material for: Factors for the development of anemia in patients with newly introduced olaparib: A retrospective case-control study
Source: Medicine (Baltimore). 2023 Jul 28;102(30):e34123. doi: 10.1097/MD.0000000000034123 (PMC10378826; doi:10.1097/MD.0000000000034123)
Supplement: Supplementary file 1 [file medi-102-e34123-s001.pdf]

Supplemental data 1. Common Terminology Criteria for Adverse Events (CTCAE) ver. 5.0

CTCAE = Common Terminology Criteria for Adverse Events, Hb = hemoglobin, LLN = lower limit of normal, TPN = total parenteral nutrition

CTCAE Term	Grade 1	Grade 2	Grade 3	Grade 4
Anemia	Hb <LLN - 10.0 g/dL;	Hb <10.0 - 8.0 g/dL	Hb <8.0 g/dL;transfusion indicated	Life-threatening consequences; urgent intervention indicated
Dysgeusia	Altered taste but no change in diet	Altered taste with change in diet (e.g., oral supplements); noxious or unpleasant taste; loss of taste	-	-
Anorexia	Loss of appetite without alteration in eating habits	Oral intake altered without significant weight loss or malnutrition; oral nutritional supplements indicated	Associated with significant weight loss or malnutrition (e.g., inadequate oral caloric and/or fluid intake); tube feeding or TPN indicated	Life-threatening consequences; urgent intervention indicated
