# Supplementary material for: Factors for the development of anemia in patients with newly introduced olaparib: A retrospective case-control study
Source: Medicine (Baltimore). 2023 Jul 28;102(30):e34123. doi: 10.1097/MD.0000000000034123 (PMC10378826; doi:10.1097/MD.0000000000034123)
Supplement: Supplementary file 2 [file medi-102-e34123-s002.pdf]

Main-study

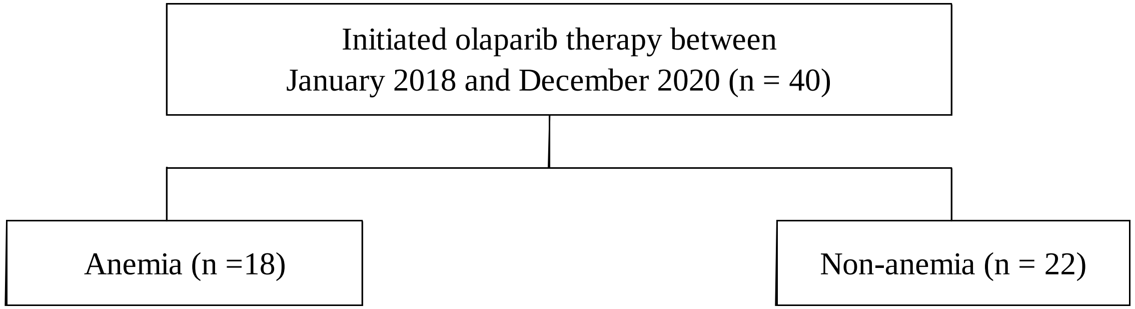

Sub-study

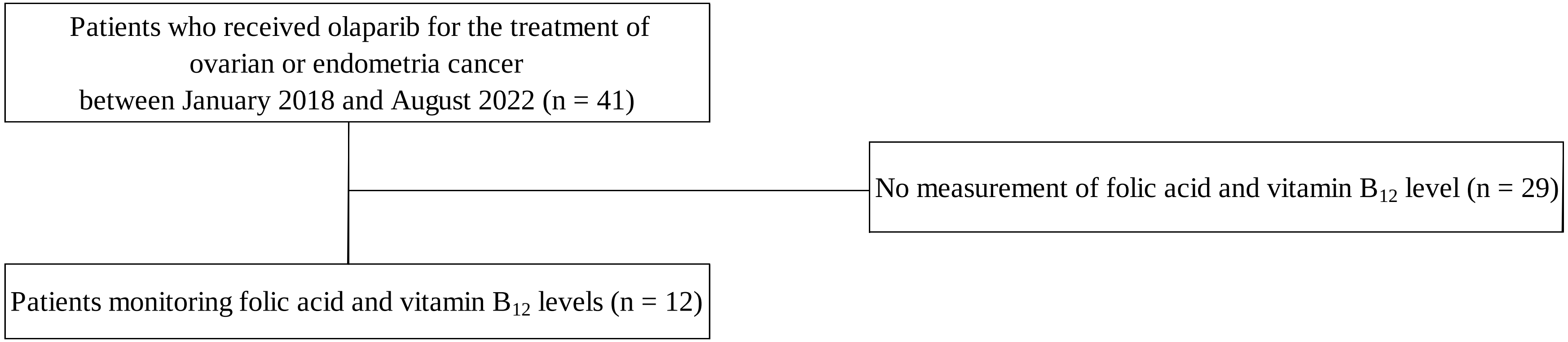

Supplemental data 2. Flowchart of the process for data collection in the main study and sub-study.

The main study included data from ovarian, breast, endometrial, prostate, and pancreatic cancer patients who were administered olaparib at Mie University Hospital between January 2018 and December 2020. We investigated the development of grade ≥3 anemia during olaparib administration for at least one year. We did not set the exclusion criteria for the preliminary analysis. During the study period, 40 patients initiated olaparib therapy and 18 (45%) patients developed grade ≥3 anemia.

The sub-study included data from patients who received olaparib for the treatment of ovarian or endometrial cancer at Mie University Hospital between January 2018 and August 2022. During the study period, 41 patients administered olaparib. Twenty-nine patients were excluded based on the following criteria: no measurement of folic acid or vitamin B_{12} levels (n = 29). Twelve patients were enrolled in the study, based on the inclusion and exclusion criteria.
