# Supplementary material for: Factors for the development of anemia in patients with newly introduced olaparib: A retrospective case-control study
Source: Medicine (Baltimore). 2023 Jul 28;102(30):e34123. doi: 10.1097/MD.0000000000034123 (PMC10378826; doi:10.1097/MD.0000000000034123)
Supplement: Supplementary file 3 [file medi-102-e34123-s003.pdf]

Supplemental data 3. Characteristics of patient's with grade ≥3 anemia

Anemia was defined as CTCAE grade ≥3 (Hb <8.0 g/dL) [21]

Alb = serum albumin, BMI = body mass index, BRCA = tumor breast cancer susceptibility gene, CTCAE = Common Terminology Criteria for Adverse Events, F = female, M = male, MCV = mean corpuscular volume, RBC = red blood cell.

| No | Sex | Age, years | BMI, $kg/m^2$ | Baseline Alb, g/dL | Baseline Hb, g/dL | Nadir Hb/ MCV | Mutated germline *BRCA*, yes | Anemia occurrence episodes, time | Initial olraparib dose, mg/day | Discontinuation /Cause | Dose reduction /Cause | RBC transfusion | Other drug treatment for anemia |
|---|---|---|---|---|---|---|---|---|---|---|---|---|---|
| 1 | M | early 70s | 27.8 | 3.0 | 7.5 | 7.0/101.3 | Yes | 1 | 300 | No | Yes/anemia | Yes | Iron |
| 2 | F | early 80s | 19.3 | 4.0 | 12.3 | 5.0/103.4 | Unknown | 1 | 400 | Yes/anemia | No | No | No |
| 3 | F | early 70s | 26.2 | 4.5 | 9.1 | 8.4/95.1 | Unknown | 1 | 400 | Yes/anemia | No | No | Iron |
| 4 | F | early 50s | 28.7 | 4.3 | 12.7 | 7.1/105.9 | Unknown | 1 | 600 | Yes/anemia | Yes/ anemia, nausea, fatigue | No | Iron |
| 5 | F | early 70s | 18.2 | 4.4 | 10.9 | 7.5/99.6 | Unknown | 1 | 600 | Yes/anemia | No | No | Iron |
| 6 | F | late 70s | 16.1 | 4.3 | 10.4 | 7.7/100.4 | Yes | 1 | 600 | Yes/anemia, fatigue | Yes/ anemia, fatigue | No | No |
| 7 | F | late 50s | 18.7 | 4.3 | 11.8 | 7.8/88.8 | Yes | 1 | 600 | Yes/anemia | No | No | Iron |
| 8 | F | late 50s | 19.6 | 3.9 | 10.0 | 7.6/96.9 | Unknown | 1 | 600 | Yes/anemia | No | No | No |
| 9 | F | late 50s | 24.7 | 4.2 | 11.1 | 7.1/94.7 | Unknown | 1 | 600 | Yes/anemia | No | Yes | No |
| 10 | F | early 60s | 20.6 | 4.0 | 11.3 | 7.3/107.4 | Yes | 2 | 600 | Yes/anemia | Yes/anemia | No | No |
| 11 | F | early 30s | 25.2 | 3.8 | 8.9 | 7.6/100.0 | Yes | 2 | 600 | Yes/anemia | Yes/anemia | No | No |
| 12 | F | early 60s | 29.4 | 4.2 | 10.4 | 7.1/108.0 | Unknown | 2 | 600 | Yes/anemia | Yes/anemia | Yes | No |
| 13 | M | late 60s | 22.7 | 4.1 | 14.4 | 7.6/115.3 | Yes | 2 | 600 | Yes/anemia, neutropenia | Yes/anemia | No | No |
| 14 | F | late 30s | 18.4 | 4.5 | 12.4 | 6.0/88.6 | Unknown | 3 | 600 | Yes/anemia | Yes/anemia | No | No |

| | | | | | | | | | | | | |
|---|---|---|---|---|---|---|---|---|---|---|---|---|
| 15 | F | early 60s | 22.0 | 4.2 | 10.1 | 7.5/97.7 | Unknown | 3 | 600 | Yes/anemia | Yes/anemia | Yes | No |
| 16 | F | early 70s | 26.0 | 4.1 | 12.3 | 5.4/96.3 | Unknown | 3 | 600 | Yes/anemia, neutropenia | Yes/anemia | Yes | No |
| 17 | F | late 50s | 23.6 | 4.4 | 13.3 | 7.6/95.0 | Unknown | 3 | 600 | Yes/anemia | Yes/anemia | No | No |
| 18 | F | early 70s | 23.0 | 4.2 | 10.5 | 7.5/117.3 | Unknown | 3 | 600 | Yes/anemia | Yes/anemia | Yes | No |
