# Supplementary material for: Factors for the development of anemia in patients with newly introduced olaparib: A retrospective case-control study
Source: Medicine (Baltimore). 2023 Jul 28;102(30):e34123. doi: 10.1097/MD.0000000000034123 (PMC10378826; doi:10.1097/MD.0000000000034123)
Supplement: Supplementary file 4 [file medi-102-e34123-s004.pdf]

Supplemental data 4. Anemia-related laboratory data of the patients at the time of initial olaparib-induced anemia

Anemia was defined as CTCAE grade ≥3 (Hb <8.0 g/dL) [21]

*Data are presented as the median [interquartile range].

ΔHb was defined as follows: ΔHb = baseline Hb level – nadir Hb level.

We categorized anemia into three groups depending on MCV (microcytic, MCV ≤80 fL; normocytic, 80 <MCV ≤100 fL; and macrocytic anemia, 100 <MCV fL) [22].

None of the patients had microcytic anemia.

CTCAE = Common Terminology Criteria for Adverse Events, Hb = hemoglobin, MCV = mean corpuscular volume, RDW-CV = red cell distribution width–coefficient of variation, RDW-SD = red cell distribution width–standard deviation.

	Anemia		*p*–value
	80 <MCV ≤100 fL (n = 9)	100 <MCV fL (n = 9)	
Anemia onset date*, day	56.0 [46.0–69.5]	70.0 [32.5–171.5]	.17
Lowest Hb value*, g/dL	6.7 [5.9–7.6]	6.8 [6.3–7.5]	.83
ΔHb*, g/dL	3.3 [2.0–6.2]	4.0 [2.5–6.1]	.76
RDW-SD*, fL	50.7 [44.4–60.0]	65.3 [55.2–72.0]	.01
RDW-CV*, %	15.2 [12.9–17.2]	17.1 [14.8–19.8]	.16
