# Supplementary material for: Factors for the development of anemia in patients with newly introduced olaparib: A retrospective case-control study
Source: Medicine (Baltimore). 2023 Jul 28;102(30):e34123. doi: 10.1097/MD.0000000000034123 (PMC10378826; doi:10.1097/MD.0000000000034123)
Supplement: Supplementary file 5 [file medi-102-e34123-s005.pdf]

Supplemental data 5. Patient characteristics based on MCV at the time of anemia.

Anemia was defined as CTCAE grade ≥3 (Hb <8.0 g/dL) [21]

The median cumulative dose of carboplatin was calculated for patients with a history of carboplatin treatment.

*Data are presented as the median [interquartile range].

Ccr = [(140 – age) × weight (kg)]/[72 × Scr (mg/dL)] (× 0.85 if female) [19]

eGFR (mL/min/1.73 m^2) = 194 × $Scr^{-1.094}$ × $age^{-0.287}$ (× 0.739 if female) [20]

Alb = serum albumin, ALT = alanine aminotransferase, AST = aspartate aminotransferase, BMI = body mass index, *BRCA* = tumor breast cancer susceptibility gene, BUN = blood urea nitrogen, CCr = creatinine clearance, CRP = C-reactive protein, CTCAE = Common Terminology Criteria for Adverse Events, eGFR = estimated glomerular filtration rate, Hb = hemoglobin, Ht = hematocrit, LDH = lactate dehydrogenase, Lym = lymphocyte, MCH = mean corpuscular hemoglobin, MCHC = mean corpuscular hemoglobin concentration, MCV = mean corpuscular volume, Net = neutrophil, Plt = platelet, RBC = red blood cell, RDW-SD = red cell distribution width standard deviation, RDW-CV = red cell distribution width coefficient of variation, Scr = serum creatinine, T-Bil = total bilirubin, WBC = white blood cell

Demographics	80 <MCV ≤100 fL (n = 9)<br>n (%)	100 <MCV fL (n = 9)<br>n (%)	*p*–value
Age*, years	58.0 [55.5–71.0]	65.0 [58.5–75.0]	.29
Female	9 (100.0)	7 (77.8)	.47
Body weight*, kg	55.4 [48.3–62.5]	61.7 [45.5–67.7]	.20
BMI*, kg/m^2	24.7 [19.7–26.0]	23.0 [19.9–28.2]	.35
History of peptic ulcer	0 (0.0)	1 (11.1)	1.00

Smoking history	3 (33.3)	4 (44.4)	1.00
Brinkman index*	170.0 [0.0–1230.0]	480 [40.8–1058.8]	.72
History of alcohol consumption	0 (0.0)	3 (33.3)	.21
Previous carboplatin treatment history	6 (66.7)	9 (100.0)	.06
Cumulative dose of carboplatin*, mg/m^2	3260.2 [2601.1–4326.4]	5090.7 [2406.2–8536.1]	.29
Radiation therapy	1 (11.1)	2 (22.2)	.56
RBC transfusion treatment	2 (22.2)	4 (44.4)	.62
**Daily olaparib dose**			
600 mg/day	8 (88.9)	8 (88.9)	1.00
400 mg/day	1 (11.1)	0 (0.0)	1.00
300 mg/day	0 (0.0)	1 (11.1)	1.00
***BRCA* mutation status**			
Mutated germline *BRCA*	7 (77.8)	5 (55.6)	.62
Unknown	2 (22.2)	4 (44.4)	.62
**Primary tumor location**			
Ovaries	8 (88.9)	6 (66.6)	.58
Breast	0 (0.0)	2 (22.2)	.47
Endometria	1 (11.1)	0 (0.0)	1.00
Prostate	0 (0.0)	1 (11.1)	1.00
Pancreas	0 (0.0)	0 (0.0)	1.00
**Clinical laboratory data**			
Alb*, g/dL	4.4 [4.2–4.5]	4.2 [4.0–4.3]	.08
Alb <4.0 g/dL	4 (44.4)	0 (0.0)	.04
CRP*, mg/dL	0.0 [0.0–0.1]	0.0 [0.0–0.2]	.78

BUN*, mg/dL	17.2 [14.6–20.0]	12.2 [10.2–15.0]	.01
Scr*, mg/dL	0.7 [0.6–0.8]	0.6 [0.5–0.7]	.09
Ccr [Cockcroft–Gault] *, mL/min	70.5 [52.2–88.2]	84.1 [66.3–104.2]	.37
eGFR*, mL/min/1.73m^2	67.0 [59.3–77.9]	80.2 [70.8–96.0]	.03
LDH*, IU/L	187.5 [163.8–228.8]	242.0 [185.0–303.0]	.11
AST*, U/L	23.0 [17.0–25.5]	22.0 [16.5–37.0]	.89
ALT*, U/L	12.0 [10.5–23.5]	13.0 [8.8–24.0]	.92
T–Bil*, mg/dL	0.5 [0.5–0.7]	0.6 [0.5–0.7]	.33
WBC*, ×10^3/μL	5.0 [4.1–5.4]	4.1 [3.4–6.3]	.45
Net*, /μL	2715.0 [2152.5–3410.0]	2855.0 [1637.5–4640.0]	.80
Lym*, /μL	1485.0 [1052.5–1570.0]	1125.0 [940.0–1607.5]	.37
Plt*, ×10^3/μL	184.0 [163.0–256.5]	174.0 [124.5–229.5]	.31
RBC*, ×10^6/μL	3.6 [3.0–3.9]	3.3 [2.8–3.8]	.40
Hb*, g/dL	11.1 [10.1–12.4]	10.5 [9.7–12.5]	.79
Ht*, %	34.3 [30.3–37.1]	32.0 [29.2–36.8]	.83
MCV*, fL	96.9 [94.8–99.5]	100.0 [97.1–101.9]	.15
MCH*, pg	32.0 [30.9–32.9]	32.5 [31.9–35.0]	.20
MCHC*, g/dL	32.7 [32.5–33.1]	33.2 [32.1–34.2]	.69
CRP/Alb ratio*, 10^{-3}	0.0 [0.0–0.1]	0.0 [0.0–0.5]	.84
RDW-SD*, fL	55.5 [48.5–64.4]	52.4 [46.7–58.5]	.57
RDW-CV*, %	17.4 [14.1–19.4]	14.8 [14.2–17.8]	.83
**Co-administered drugs**			
Suppressing folic acid	0 (0.0)	0 (0.0)	1.00
Suppressing vitamin B_{12}	1 (11.1)	1 (11.1)	1.00
